# Quantitative Analysis and Fingerprint Profiles for Quality Control of Fructus Schisandrae by Gas Chromatography: Mass Spectrometry

**DOI:** 10.1155/2014/806759

**Published:** 2014-01-12

**Authors:** Yong-Gang Xia, Bing-You Yang, Jun Liang, Qi Yang, Di Wang, Hai-Xue Kuang

**Affiliations:** Key Laboratory of Chinese Materia Medica, Heilongjiang University of Chinese Medicine, Ministry of Education, Harbin 150040, China

## Abstract

This paper describes a simple, rapid, and effective quality assessment method for Fructus Schisandrae by gas chromatography-mass spectrum (GC-MS). The method was established by using specific lignan fingerprint profiles and quantitation of characteristic compounds in this herbal medicine. The GC-MS fingerprints of 15 batches of *Schisandra* samples from different regions of China showed similar lignan profiles. Five peaks were selected as characteristic peaks, and all of these were identified by using GC-MS techniques. The relative retention times of these characteristic peaks in the GC-MS fingerprint were established as an important parameter for identification of *Schisandra* samples. Meanwhile, relative peak areas may be a feasible approach to discriminate the *S. chinensis* and *S. sphenanthera*. Finally, these pharmacologically active constituents in the titled plant, schisandrins A–C and schizandrols A and B, were quantitatively determined using a validated GC-MS method.

## 1. Introduction

Traditional Chinese Medicines (TCMs) have made great contribution to the health of Chinese people for thousands of years due to its special clinical efficacy [1]. Gradually, TCMs have been attracting interest and acceptance in many western countries. This may be primarily because of the general belief that herbal drugs are without any side effect besides being cheap and locally available [2]. However, the quality of TCMs had an important influence on its clinical efficacy due to their different species, harvest season, storage, and geographic origins [3]. Therefore, quality control of TCMs is becoming extremely urgent. There is no denying the fact that multicomponents of TCMs are responsible for the therapeutic effects by the multipath-assisted multitarget approach. Therefore, in recent years, there is an increasing trend to establish multicomponents analysis for the effective quality control of TCMs [4–6].

Fructus Schisandrae, the ripe fruits of *Schisandra sphenanthera* Rehd. et Wils and *S. chinensis *(Turcz.) (Schisandraceae), is one of the most famous TCMs and has been widely used for thousands of years in China. Since the 2000 edition of Chinese Pharmacopoeia, the fruits of *S. sphenanthera* and *S. chinensis* have been accepted as two different crude drugs, “Nan-wuweizi” (Fructus Schisandrae Sphenanthera) and “Bei-wuweizi” (Fructus Schisandrae Chinensis) [7], which distributed in the southern provinces (Shanxi, Shaanxi, Gansu, Shandong, Jiangsu, etc.) and northern provinces of China (Heilongjiang, Jilin, Liaoning, Neimenggu, etc.), respectively. Wuweizi has been utilized as a sedative and tonic to treat various diseases such as chronic cough and dyspnea, nocturnal emission, spermatorrhea, enuresis, and frequent urination and could also be used as flavouring agent of foods [8]. Moreover, many Chinese medicinal preparations containing Wuweizi or its extract are widely used in China, such as “Hugan tablet,” “Jiangtang pills,” “Compound Schisandra Syrup,” “Shenqi Wuweizi tablet,” and “Shengmaiyin,” [9, 10].

Modern pharmacological research has demonstrated that most of the biological actions and the pharmacological effects of Wuweizi can be attributed to its dibenzocyclooctadiene-type lignans, which have been elucidated to play an important role in antioxidant, anti-inflammatory, anxiolytic effect, protective hepatic injury, acetylcholinesterase inhibitory effect, and stimulation of the carbohydrate-phosphorus metabolism, and so forth [8, 11–14]. Thus, quantitative analysis of multiple-lignans in Fructus Schisandrae is very essential to control its quality. In terms of quantitative analysis of *S. sphenanthera* and *S. chinensis*, many analytical methods have been reported for the determinations of lignans, including HPLC with UV detection, HPLC with mass spectrum (HPLC-MS), and capillary electrophoresis (CE) [9, 15–20]. Despite the fact that HPLC and CE coupled with UV detector are very useful for the determination of lignans in *Schisandra* sample, they are powerless to be used for identifying constituents. Additionally, HPLC-MS instruments are still inaccessible for most of laboratories worldwide.

Though gas chromatography-mass spectrometry (GC-MS) is commonly used to determine the volatile components of herb medicines, it has been successfully applied to detect some thermally stable lignans in essential oil fractions of *S. chinensis* with supercritical fluid extraction [21–23]. Moreover, as far as our knowledge, there are no reports of the application of GC-MS in assays dedicated to study quality control of *S. chinensis *and *S. sphenanthera* by lignan chromatography profiles. To establish an improved quality assessment method for Fructus Schisandrae, such a fundamental truth promotes our interests and encourages us to consider applying GC-MS fingerprint profiles combined with quantification of multi-ingredients for quality evaluation of Fructus Schisandrae. Due to the powerful separation efficiency and the sensitive detection, GC-MS has become a popular and useful analytical tool in the research field of herbal medicines [24].

In the present study, GC-MS fingerprint profiles can provide sufficient qualitative information for the identification and authentication of Fructus Schisandrae by ultrasound-assisted extraction (UAE). Five active components, namely, schisandrins A–C (**1**–**3**) and schizandrols A and B (**4 **and **5**), which were the major chemical constituents in the fingerprint with known biological activities, were selected for simultaneous quantification. The newly established method was utilized to analyze 15 samples collected from different regions of China. The development of this new simple and fast methodology for *Schisandra* lignan analysis provides a valuable tool to evaluate quality of traditional Chinese medicine Wuweizi.

## 2. Experimental

### 2.1. Chemicals and Materials

**Figure 1:**
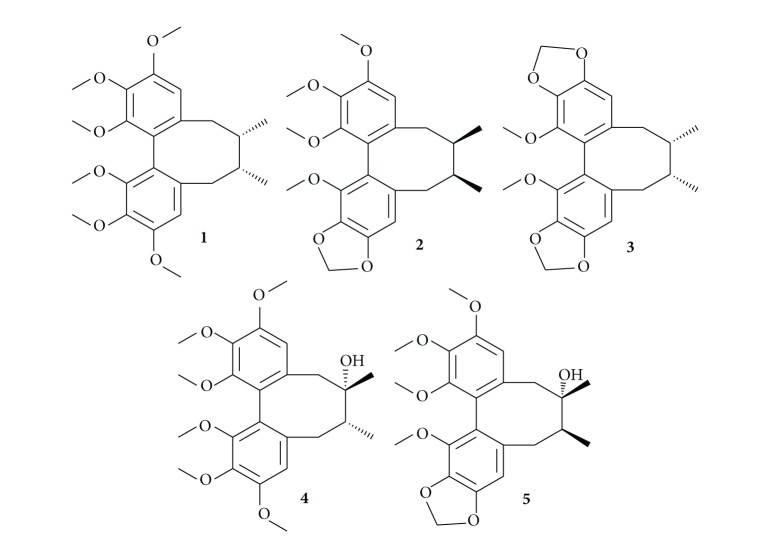
Structures of schisandrin A (**1**), schisandrin B (**2**), schisandrin C (**3**), schisandrol A (**4**), and schisandrol B (**5**).

Standards of schisandrin A (**1**), schisandrin B (**2**), schisandrin C (**3**), schisandrol A (**4**), and schisandrol B (**5**) were purchased from the Chengdu JSMT Biotechnology Co. Ltd. (Chengdu, China). Their structures can be seen in Figure 1. Samples 1–4 were collected on September 2010 as raw materials from Daxinganling District, Heilongjiang, China. Sample 5 was collected on October 2011 from medical botany park of Heilongjiang University of Chinese Medicine. Samples 6–8 were collected on September 2010 as raw materials from Fangzhen, Raohe, and Qitaihe districts, Heilongjiang province, China. Samples 9–15 were collected as decoction pieces on December 2010 form Harbin medical market. Detailed description of samples was listed in Table 4. HPLC grade methanol (MeOH) was purchased from Dikama Technology Corporation (Richmond Hill, USA). All other reagents were of analytical grade.

### 2.2. GC-MS Apparatus and Conditions

The analyses were performed using an Agilent 7890A–5975C instrument equipped with a DB-17 fused-silica capillary column (60 m × 0.25 mm × 0.25 um) and an Agilent 5975C MS detector. One microlitre of the sample was injected into GC-MS using split mode (5 : 1). The purge flow was 1.2 mL/min. The injector temperature was 250°C. The operation was performed at a column temperature program from 120°C to 250°C at 10°C/min, then increasing to 280°C at 5°C/min and finally holding for 30 min at 280°C. All data were obtained by collecting the full-scan mass spectra within the scan range of 40–600 amu.

### 2.3. Preparation of Sample Solutions

The dried powders of *Schisandra *samples (0.25 g, 60 mesh) were accurately weighed and extracted by ultrasonic with 10 mL methanol solution for 40 min at 60°C and 70 kHz under ultrasonic irradiation. Then, the resultant mixture was adjusted to the original weight with methanol and the supernatant was filtered through 0.22 *μ*m membrane before GC-MS analysis.

### 2.4. Method Validation

A methanol stock solution containing all 5 reference standards was prepared by dissolving the reference standards in methanol to a final concentration of 0.40 mg/mL for each reference standard, then diluted the mixture stock solution to appropriate concentration to establish calibration curves. Each calibration curve concentration was performed in triplicate. All calibration curves were constructed from one ten thousandth of peak areas of reference standards (*A* × 10^−4^) versus their concentrations (*c*, mg/mL). The lowest concentration of working solution was diluted with methanol to yield a series of appropriate concentrations, and the LOD and LOQ under the chromatographic conditions were separately determined at an *S*/*N* of 3 and 10, respectively. The measurement of intra- and interday variability was utilized to determine the precision of this newly developed method. The intraday variation was determined by analyzing the same mixed standard methanol solution for six times within 1 day. While for interday variability test, the solution was examined in triplicate for 3 consecutive days.

## 3. Results and Discussion

### 3.1. Optimization of Sample Extraction Conditions

In order to obtain quantitative extraction, UAE was optimized with methanol as an extract solvent. Ultrasonic technique is being used widely in analytical chemistry, facilitating different steps in the analytical process, particularly in sample preparation. UAE is an expeditious, inexpensive, and efficient alternative to traditional extraction techniques [25]. The variables involved in the procedure such as volume of methanol, extraction time, sonication frequency, and extraction temperature were investigated by using 0.25 g plant sample. Effects of the solvent volume (10; 20; 30; 40; 50 mL) on the extracting yield were tested with different volumes of methanol and the extraction for 15 min at 40°C and 60 kHz. The maximum yield was obtained at 10 mL. Normally, the usage of larger volume of solvent for extraction is able to obtain higher yields. However, this result was special, and the same circumstance was found for ultrasound-assisted extraction of phillyrin from *Forsythia suspensa* [26].

Extraction time had a close relationship with extraction efficiency. In the assay, extraction efficiency in samples was compared by sonication with 10 mL of methanol at 40°C and 60 kHz for 10, 15, 20, 30, 40, and 50 min, respectively. The results indicated that the highest extraction efficiency was obtained by sonication for 40 min in pure methanol. In this study, effects of different temperatures (30, 40, 50, 60, and 70°C) and sonication frequencies (40, 50, 60, 70, and 80 kHz) on the extracting yield were also investigated. By comparing peak areas of the five investigated components, it was found that, when 60°C and 70 kHz was employed, the peak areas of the five investigated components reached the highest values.

From the above experiments, it was demonstrated that the most suitable UAE condition for lignans from Fructus Schisandrae was 0.25 g plant sample with 10 mL of methanol and the extraction for 40 min at 60°C and 70 kHz under ultrasonic irradiation.

### 3.2. Optimization of GC-MS Conditions

Optimization of GC-MS parameters was done through investigating the influence of the column, temperature program, and split ratio on the information content. Capillary columns OV-17, DB-17, and DB-5 were screened. DB-17 column showed higher resolution and shorter analysis time than those obtained on the other two columns. Temperature program and split ratio were also studies. Finally, the optimized GC-MS analysis condition was developed for specific analysis of lignans in Fructus Schisandrae, as stated in instrumentation and conditions section above.

### 3.3. GC-MS Fingerprint Identification and Discrimination

**Figure 2:**
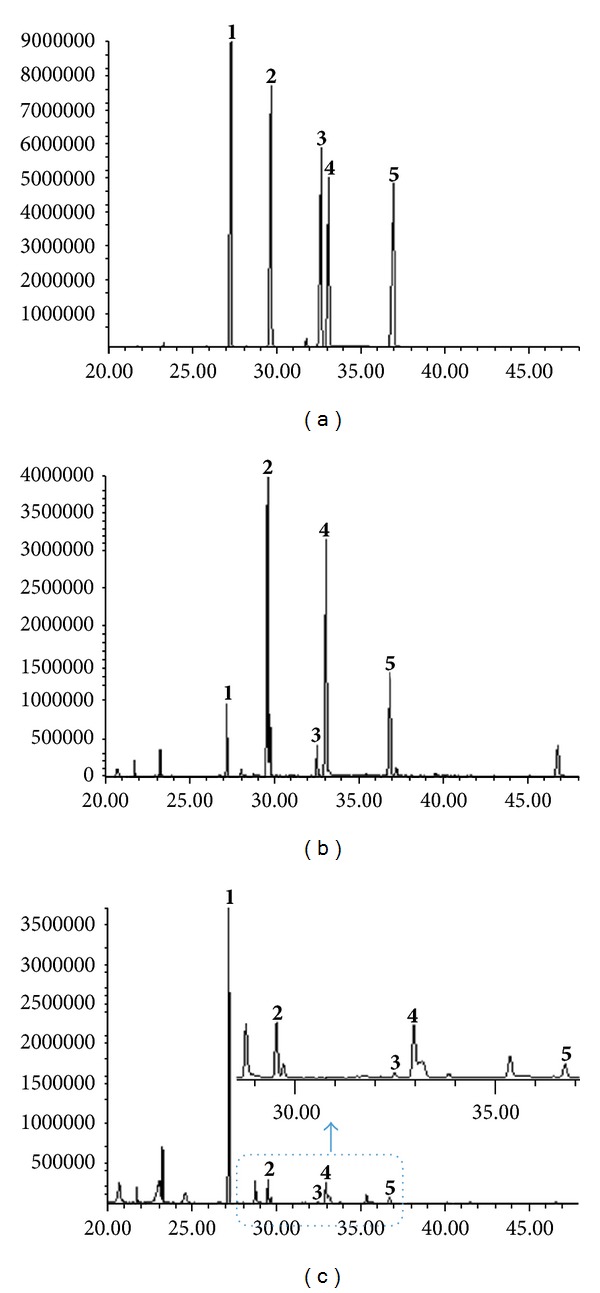
Typical GC-MS fingerprinting chromatograms. (a) Mix standards; (b) *S. chinensis *(Gaoleng, Daxinganling, Heilongjiang); (c) *S. sphenanthera* (Henan). The retention time is defined as the minute. The structures of marker peaks **1**–**5** can be seen in Figure 1.

Though a number of lignan constituents have to be derivatization for detection by GC-MS [27, 28], some specific dibenzocyclooctadiene-type lignans in Fructus Schisandrae were directly detected by GC-MS without derivatization. Figure 2 showed three typical fingerprint profiles of standards *S. chinensis* and *S. sphenanthera* under the optimized UAE and GC-MS conditions. There are many terpenes and fatty acid derivatives in Wuweizi samples before 20 minutes, which were in agreement with previous studies [21]. However, at present study, we focused on lignan components for separation and detection.

The GC-MS fingerprint profiles of 15 batches of samples were obtained from different regions of China. These samples showed similar lignan profiles after 20 minutes. By carefully analyzing the fingerprint profiles of these samples, five interest peaks were selected as characteristic peaks for the identification of the crude drugs originating from *S. chinensis* and *S. sphenanthera*. Peak 1 was selected as the marker peak due to acceptable heights and good resolution. Relative retention times (RRTs) and relative peak areas (RPAs) of the five characteristic peaks were calculated as follows: RRT = retention time of characteristic peak/retention time of marker peak, and RPA = peak area of characteristic peak/peak area of marker peak.

GC-MS was further used to identify the chemical constituents of the *Schisandra* lignans.  Table 1 lists the retention times (*t*_*R*_) and MS data of five interest peaks. The mass spectra matched with those obtained for the pure standards for each of the components of interest **1**–**5**, thus confirming their identity. As listed in Table 1, these five components exhibited their quasi-molecular ions [M]^+^. Their fragmentation patterns are well matched with their chemical structures [22]. Thus, five interest peaks (**1**–**5**) in the GC-MS fingerprint profile were unambiguously identified as schisandrin A (**1**), schisandrin B (**2**), schisandrin C (**3**), schisandrol A (**4**), and schisandrol B (**5**), respectively. According to the *m*/*z* values and retention features, the five components were identified from methanol extract of 15 batches of Wuweizi samples.

RPAs of the five characteristic peaks varied dramatically (RSD% ≥ 93.817), but the RRTs showed excellent consistency (RSD% ≤ 0.098) (Table 2). Thus, RRT should be a suitable parameter for identification of *Schisandra* samples. Therefore, a sample with a similar GC-MS lignan profile and matched RRT values (Table 2) to the typical fingerprint chromatogram shown in Figures 2(b) and 2(c) can be authenticated as genuine *S. chinensis* and *S. sphenanthera*. Furthermore, RPA seems to be a suitable parameter for discrimination of* Schisandra* samples, especially peak ratios between schisandrin B (**2**) and schisandrin A (**1**) and between schisandrol A (**3**) and schisandrin A (**1**). As can be seen from Table 2, *S. chinensis* samples (1–11) produced RPA value of **2**/**1** that ranged from 0.7 to 5.5, but *S. sphenanthera* (12–15) samples produced the ratios of **2**/**1** which is less than 0.1. Similarly, the former had much higher rations of **4**/**1** from 1.1 to 11.0 than those of the later of less than 0.12. The results indicated that it may be a feasible approach to discriminate the *S. chinensis* and* S. sphenanthera* by the RPA values of **2**/**1 **and** 4**/**1**. However, our hypothesis should be further confirmed by testing more *Schisandra* samples.

### 3.4. Quantitative Determination

#### 3.4.1. Method Validation

As shown in Table 3, all calibration curves showed good linear regression (*R* ^2^ ≥ 0.9990) within the test ranges. The LOD (*S*/*N* = 3) and the LOQ (*S*/*N* = 10) were less than 0.063 and 0.185 *μ*g/mL for all analytes. In many cases of GC/MS analysis, the LOD could be greatly decreased by adjusting the sample volume, the detection mode, such as scan or selected ion monitoring, and the injection mode [29]. The intraday and interday precisions were less than 1.29%. A recovery study was performed to validate the accuracy of the developed method. Sample 2 (0.25 g) was spiked with different levels (50, 100, and 150%) of known amounts of the compounds **1**–**5**. The spiked samples were extracted with 10 mL methanol following the procedure for sample preparation as described above. The recovery was determined by comparing the amount of analyte added to the sample and the amount of analyte detected during GC-MS analysis. As shown in Table 4, the developed analytical method provided good accuracy with the recoveries from 96.36 to 105.42%, with RSDs of less than 2.14% for the analytes. Hence, this verified GC-MS method was precise, accurate, and sensitive for the quantitative evaluation of major active components in *S. chinensis* and *S. sphenanthera*.

#### 3.4.2. Sample Analysis

A number of pharmacological activities of these five components were previously reported [12–14, 30]. Qualitative and quantitative analysis of these characteristic constituents could play an important role in evaluating and controlling the quality of *Schisandra* samples. The developed GC-MS method was then successfully applied to simultaneously determine the five components in 15 batches of *Schisandra* samples obtained from different species, geographic origin, and source.

The results showed that there were remarkable differences in the contents of the five compounds in 15 batches of *Schisandra* samples (Table 5). The total content of the five lignans changed from 7.77 to 15.513 mg/g which was found in *S. chinensis*. However, the contents of these lignans were less than 6.6 mg/g in *S. sphenanthera*. Among the five lignans, the contents of schisandrol A and schisandrin B were higher in *S. chinensis* than those from *S. sphenanthera*. However, the contents of schisandrin A were higher in *S. sphenanthera *than those from *S. chinensis*, which were in agreement with previous studies [31]. These variations might be on account of the different species, plant origins, harvesting time, storage conditions, and so forth. The variation in contents of active components may cause changes in clinical efficacy. So, our results further confirmed that it is reasonable to classify fruits of *S. sphenanthera* and *S. chinensis* as two different crude drugs since the 2000 edition of Chinese Pharmacopoeia [7].

**Figure 3:**
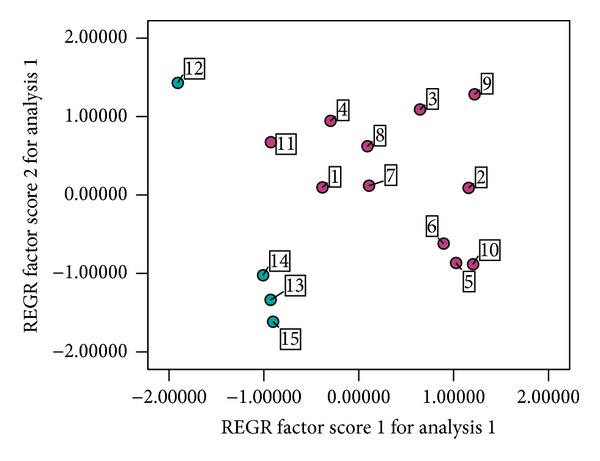
Score plots from PCA. Numbers 1–11 are samples of *S. chinensis* and numbers 11–15 are samples of* S. sphenanthera*.

This recognition can be further confirmed by principal-components projection analysis (PCA) using the contents of the 5 characteristic compounds that were performed on the analytical data of all 15 samples. Obviously, except for sample 12, other tested samples apparently form into two big clusters according to different species, the *S. chinensis* cluster and *S. sphenanthera *cluster (Figure 3), indicating that a global chemical difference was present between the two species. However, within the same species, the clustering was not very closely packed and some individual samples were sparsely distributed. This also implies that plant origins, harvesting time, processing, and storage conditions will have an important influence on their qualities.

## 4. Conclusions

There is to date no reports for combining characteristic GC-MS fingerprint profiles with quantitation of multiple-lignans for quality control of Fructus Schisandrae. GC-MS is used to construct characteristic fingerprint profiles for recognition for specific lignans in Fructus Schisandrae. This method can not only give an overview of all the specific lignans detected in Fructus Schisandrae, but also quantitate some active constituents. Thus, this approach can be applied to control the quality of Fructus Schisandrae effectively. We expected that GC-MS fingerprint profiles with quantitation of multiple-lignans can be used as an effective alternative quality assessment model for Fructus Schisandrae.

## Figures and Tables

**Table 1:** Identification of the five compounds by GC-MS. The structures of **1–5** can be seen in Figure 1.

No.	*t*_*R*_ (min)	EI-MS fragments, *m/z* (abundance %).
**1**	27.21	416 (100), 370 (7), 330 (7), 285 (5), 235 (6), 201 (1), 157 (2), 115 (1), 77 (1), 41 (1)

**2**	29.63	400 (100), 354 (4), 312 (7), 272 (3), 235 (3), 165 (3), 128 (2), 77 (1), 41 (1)

**3**	32.55	384 (100), 328 (11), 283 (7), 219 (8), 165 (5), 115 (4), 77 (1), 41 (1)

**4**	33.09	432 (100), 389 (12), 330 (63), 287 (8), 241 (3), 180 (10), 128 (3), 91 (1), 43 (5)

**5**	36.87	416 (100), 373 (9), 341 (75), 271 (9), 215 (5), 172 (11), 128 (6), 91 (2), 43 (7)

**Table 2:** Relative retention times (RRTs) and relative peak areas (RPAs) of five characteristic peaks in GC-MS fingerprints of 15 batches of *Schisandra* samples.

No.	**1**		**2**		**3**		**4**		**5**	
	RRT	RPA	RRT	RPA	RRT	RPA	RRT	RPA	RRT	RPA
1	1.000	1.000	1.089	5.488	1.196	0.548	1.216	5.584	1.355	2.546
2	1.000	1.000	1.088	2.956	1.196	0.783	1.215	3.895	1.354	1.407
3	1.000	1.000	1.087	3.078	1.196	0.938	1.216	10.368	1.355	4.876
4	1.000	1.000	1.087	1.315	1.196	0.137	1.215	3.417	1.353	0.725
5	1.000	1.000	1.087	0.956	1.195	0.059	1.215	1.711	1.352	0.277
6	1.000	1.000	1.087	1.035	1.195	0.061	1.215	1.815	1.352	0.342
7	1.000	1.000	1.088	3.716	1.196	0.653	1.215	3.995	1.354	1.886
8	1.000	1.000	1.088	3.225	1.196	0.627	1.216	5.756	1.354	1.859
9	1.000	1.000	1.088	3.704	1.196	0.447	1.216	11.037	1.355	5.048
10	1.000	1.000	1.087	1.059	1.195	0.058	1.215	1.856	1.352	0.332
11	1.000	1.000	1.087	0.721	1.195	0.087	1.214	1.135	1.352	0.321
12	1.000	1.000	1.087	0.033	1.198	0.000	1.216	0.003	1.353	0.000
13	1.000	1.000	1.086	0.065	1.197	0.027	1.216	0.105	1.357	0.027
14	1.000	1.000	1.086	0.080	1.196	0.031	1.212	0.083	1.352	0.046
15	1.000	1.000	1.085	0.038	1.196	0.007	1.214	0.039	1.353	0.003
RSD%	0.00	0.00	0.092	93.817	0.063	110.860	0.098	104.506	0.098	128.425

Note: numbers 1–11 are samples of *S. chinensis* and numbers 11–15 are samples of* S. sphenanthera.* The structures of **1–5 **can be seen in Figure 1.

**Table 3:** Calibration parameters of GC-MS analysis for the 5 compounds. The structures of **1**–**5** can be seen in Figure 1.

No.	Regression equations	Linear range (*μ*g/mL)	*R* ^2^	LODs (*μ*g/mL)	LOQs (*μ*g/mL)
**1**	*y* = 123574*x* + 1644.7	6.25–400.0	0.9990	0.05	0.15
**2**	*y* = 137292*x* + 879.15	6.25–400.0	0.9995	0.05	0.15
**3**	*y* = 110898*x* + 454.5	6.25–400.0	0.9997	0.063	0.185
**4**	*y* = 90690*x* + 63.81	6.25–400.0	0.9994	0.063	0.185
**5**	*y* = 110631*x* + 172.72	6.25–400.0	0.9991	0.063	0.185

**Table 4:** Recovery experiment of analytical method for five components.

No.	Original (mg)	Spiked (mg)	Found (mg)	Mean recovery (%)	RSD (%) (*n* = 3)
**1**	0.186	0.110	0.292	96.36	1.72
		0.220	0.413	103.18	1.53
		0.330	0.505	96.67	1.47

**2**	0.225	0.173	0.407	105.20	2.14
		0.346	0.573	100.58	1.63
		0.519	0.755	102.12	1.27

**3**	0.174	0.220	0.388	97.27	1.39
		0.240	0.427	105.42	1.52
		0.460	0.623	97.61	2.08

**4**	2.326	2.320	4.756	104.74	1.58
		4.640	6.916	98.92	1.33
		6.960	9.176	98.42	1.79

**5**	0.893	0.490	1.413	106.12	1.65
		0.980	1.855	98.16	1.34
		1.470	2.406	102.93	1.27

**Table 5:** The measurement results of marker compounds in *S. chinensis* and *S. sphenanthera* (mg/g). The structures of **1–5** can be seen in Figure 1.

No.	Source	Geographical regions	**1**	**2**	**3**	**4**	**5**
1	*S. chinensis *	Tahe, Heilongjiang	0.709	0.626	0.971	6.270	1.905
2	*S. chinensis *	Gaoheng, Heilongjiang	0.744	0.899	0.695	9.305	3.573
3	*S. chinensis *	Huma, Heilongjiang	0.191	0.508	0.587	9.677	3.718
4	*S. chinensis *	Jiagedaqi, Heilongjiang	0.723	0.269	0.043	5.735	1.000
5	*S. chinensis *	Harbin, Heilongjiang	2.921	0.648	0.098	7.957	1.295
6	*S. chinensis *	Fangzhen, Heilongjiang	2.564	0.619	0.049	7.676	1.139
7	*S. chinensis *	Raohe, Heilongjiang	0.636	0.772	0.693	5.756	2.335
8	*S. chinensis *	Qitaihe, Heilongjiang	0.450	0.532	0.587	7.773	2.082
9	*S. chinensis *	Liaoning	0.213	0.520	0.229	10.589	3.962
10	*S. chinensis *	Neimeng	2.814	0.706	0.063	8.444	1.229
11	*S. chinensis *	Neimeng	1.576	0.081	0.058	3.347	0.747
12	*S. sphenanthera *	Shanxi	0.274	tr.	0.000	tr.	0.000
13	*S. sphenanthera *	Hubei	4.935	0.064	0.002	0.754	0.103
14	*S. sphenanthera *	Henan	4.319	0.099	0.001	0.423	0.189
15	*S. sphenanthera *	Sichuan	5.610	tr.	tr.	0.799	0.170

Note: tr. means trace amount with less than 0.001.

## References

[1] Jiang M, Zhang C, Cao H, Chan K, Lu A (2011). The role of Chinese medicine in the treatment of chronic diseases in China. *Planta Medica*.

[2] Li SP, Zhao J, Yang B (2011). Strategies for quality control of Chinese medicines. *Journal of Pharmaceutical and Biomedical Analysis*.

[3] Xia YG, Yang BY, Wang QH, Liang J, Wang D, Kuang HX (2013). Species classification and quality assessment of Cangzhu (Atractylodis Rhizoma) by high-performance liquid chromatography and chemometric methods. *Journal of Analytical Methods in Chemistry*.

[4] Liu E-H, Qi L-W, Li K, Chu C, Li P (2010). Recent advances in quality control of traditional Chinese medicines. *Combinatorial Chemistry and High Throughput Screening*.

[5] Xie P-S, Leung AY (2009). Understanding the traditional aspect of Chinese medicine in order to achieve meaningful quality control of Chinese materia medica. *Journal of Chromatography A*.

[6] Jiang Y, David B, Tu P, Barbin Y (2010). Recent analytical approaches in quality control of traditional Chinese medicines-a review. *Analytica Chimica Acta*.

[7] State-Pharmacopoeia-Committee (2010). *Pharmacopoeia of People's Republic of China*.

[8] Panossian A, Wikman G (2008). Pharmacology of Schisandra chinensis Bail.: an overview of Russian research and uses in medicine. *Journal of Ethnopharmacology*.

[9] Zhang H, Zhang G, Zhu Z (2009). Determination of six lignans in Schisandra chinensis (Turcz.) Baill. Fruits and related Chinese multiherb remedies by HPLC. *Food Chemistry*.

[10] Yang L-Q, Wu X-Y, Xu Z-Q, Hou H-R, Fu H-Z (2005). Research progress on determination of lignans from Schisandra chinensis and its preparations. *China Journal of Chinese Materia Medica*.

[11] Chen W-W, He R-R, Li Y-F, Li S-B, Tsoi B, Kurihara H (2011). Pharmacological studies on the anxiolytic effect of standardized Schisandra lignans extract on restraint-stressed mice. *Phytomedicine*.

[12] Ci X, Ren R, Xu K (2010). Schisantherin a exhibits anti-inflammatory properties by down-regulating NF-*κ*B and MAPK signaling pathways in lipopolysaccharide-treated RAW 264.7 cells. *Inflammation*.

[13] Tran MH, Na M, Byung SM (2007). Acetylcholinesterase inhibitory effect of lignans isolated from Schizandra chinensis. *Archives of Pharmacal Research*.

[14] Pao TT, Hsu KF, Liu KT, Chang LG, Chuang CH, Sung CY (1977). Protective action of schisandrin B on hepatic injury in mice. *Chinese Medical Journal*.

[15] Lee HJ, Kim CY (2010). Simultaneous determination of nine lignans using pressurized liquid extraction and HPLC-DAD in the fruits of Schisandra chinensis. *Food Chemistry*.

[16] Gao S, You J, Wang Y, Zhang R, Zhang H (2012). On-line continuous sampling dynamic microwave-assisted extraction coupled with high performance liquid chromatographic separation for the determination of lignans in Wuweizi and naphthoquinones in Zicao. *Journal of Chromatography B*.

[17] Halstead CW, Lee S, Khoo CS, Hennell JR, Bensoussan A (2007). Validation of a method for the simultaneous determination of four schisandra lignans in the raw herb and commercial dried aqueous extracts of Schisandra chinensis (Wu Wei Zi) by RP-LC with DAD. *Journal of Pharmaceutical and Biomedical Analysis*.

[18] Kvasničková L, Glatz Z, Štěrbová H, Kahle V, Slanina J, Musil P (2001). Application of capillary electrochromatography using macroporous polyacrylamide columns for the analysis of lignans from seeds of Schisandra chinensis. *Journal of Chromatography A*.

[19] He X-G, Lian L-Z, Lin L-Z (1997). Analysis of lignan constituents from Schisandra chinensis by liquid chromatography-electrospray mass spectrometry. *Journal of Chromatography A*.

[20] Huang X, Song F, Liu Z, Liu S (2007). Studies on lignan constituents from Schisandra chinensis (Turcz.) Baill. fruits using high-performance liquid chromatography/electrospray ionization multiple-stage tandem mass spectrometry. *Journal of Mass Spectrometry*.

[21] Schwarzinger C, Kranawetter H (2004). Analysis of the active compounds in different parts of the Schisandra chinensis plant by means of pyrolysis-GC/MS. *Monatshefte fur Chemie*.

[22] Xiang Z, Li H, Zhang L (2003). Study on supercritical carbon dioxide extract from schisandra chinensis by gas chromatography-mass spectrometry. *Chinese Journal of Chromatography*.

[23] Wang L, Chen Y, Song Y, Chen Y, Liu X (2008). GC-MS of volatile components of Schisandra chinensis obtained by supercritical fluid and conventional extraction. *Journal of Separation Science*.

[24] Ruan G-H, Li G-K (2007). The study on the chromatographic fingerprint of Fructus xanthii by microwave assisted extraction coupled with GC-MS. *Journal of Chromatography B*.

[25] Lianfu Z, Zelong L (2008). Optimization and comparison of ultrasound/microwave assisted extraction (UMAE) and ultrasonic assisted extraction (UAE) of lycopene from tomatoes. *Ultrasonics Sonochemistry*.

[26] Xia E-Q, Ai X-X, Zang S-Y, Guan T-T, Xu X-R, Li H-B (2011). Ultrasound-assisted extraction of phillyrin from Forsythia suspensa. *Ultrasonics Sonochemistry*.

[27] Bonzanini F, Bruni R, Palla G, Serlataite N, Caligiani A (2009). Identification and distribution of lignans in Punica granatum L. fruit endocarp, pulp, seeds, wood knots and commercial juices by GC-MS. *Food Chemistry*.

[28] Sedlák É, Boldizsár I, Borsodi L (2008). Identification and quantification of lignans, carboxylic acids and sugars in the leaves of Forsythia species and cultivars. *Chromatographia*.

[29] Chun M-H, Kim EK, Yu SM (2011). GC/MS combined with chemometrics methods for quality control of Schizonepeta tenuifolia Briq: determination of essential oils. *Microchemical Journal*.

[30] Fong W-F, Wan C-K, Zhu C-Y (2007). Schisandrol A from Schisandra chinensis reverses P-glycoprotein-mediated multidrug resistance by affecting Pgp-substrate complexes. *Planta Medica*.

[31] Zhou Y, Huang S-X, Pu J-X (2011). Ultra performance liquid chromatography coupled with quadrupole time-of-flight mass spectrometric procedure for qualitative and quantitative analyses of nortriterpenoids and lignans in the genus Schisandra. *Journal of Pharmaceutical and Biomedical Analysis*.

